# Glutamate-glutamine homeostasis is perturbed in neurons and astrocytes derived from patient iPSC models of frontotemporal dementia

**DOI:** 10.1186/s13041-020-00658-6

**Published:** 2020-09-14

**Authors:** Blanca I. Aldana, Yu Zhang, Pia Jensen, Abinaya Chandrasekaran, Sofie K. Christensen, Troels T. Nielsen, Jørgen E. Nielsen, Poul Hyttel, Martin R. Larsen, Helle S. Waagepetersen, Kristine K. Freude

**Affiliations:** 1 Department of Drug Design and Pharmacology, Faculty of Health and Medical Sciences, University of Copenhagen, Universitetsparken 2, 2100 Copenhagen, Denmark; 2 Department of Veterinary Clinical and Animal Sciences, Faculty of Health and Medical Sciences, University of Copenhagen, Grønnegårdsvej 7, 1870 Frederiksberg C, Denmark; 3 Department of Biochemistry and Molecular Biology, University of Southern Denmark, 5230 Odense M, Denmark; 4 Danish Dementia Research Center, Department of Neurology, Rigshospitalet, University of Copenhagen, 2100 Copenhagen, Denmark; 5 Current Address: Department of Experimental Medical Science, Wallenberg Neuroscience Center and Lund Stem Cell Center, Lund University, 22184 Lund, Sweden

**Keywords:** FTD3, CHMP2B, iPSC-derived neuron, Glucose metabolism, Glutamate, Glutamine, GC-MS, GDH, PAG, GS

## Abstract

**Abstract:**

Frontotemporal dementia (FTD) is amongst the most prevalent early onset dementias and even though it is clinically, pathologically and genetically heterogeneous, a crucial involvement of metabolic perturbations in FTD pathology is being recognized. However, changes in metabolism at the cellular level, implicated in FTD and in neurodegeneration in general, are still poorly understood. Here we generate induced human pluripotent stem cells (hiPSCs) from patients carrying mutations in *CHMP2B* (FTD3) and isogenic controls generated via CRISPR/Cas9 gene editing with subsequent neuronal and glial differentiation and characterization. FTD3 neurons show a dysregulation of glutamate-glutamine related metabolic pathways mapped by ^13^C-labelling coupled to mass spectrometry. FTD3 astrocytes show increased uptake of glutamate whilst glutamate metabolism is largely maintained. Using quantitative proteomics and live-cell metabolic analyses, we elucidate molecular determinants and functional alterations of neuronal and glial energy metabolism in FTD3. Importantly, correction of the mutations rescues such pathological phenotypes. Notably, these findings implicate dysregulation of key enzymes crucial for glutamate-glutamine homeostasis in FTD3 pathogenesis which may underlie vulnerability to neurodegeneration.

**Graphical abstract:**

Neurons derived from human induced pluripotent stem cells (hiPSCs) of patients carrying mutations in CHMP2B (FTD3) display major metabolic alterations compared to CRISPR/Cas9 generated isogenic controls. Using quantitative proteomics, ^13^C-labelling coupled to mass spectrometry metabolic mapping and seahorse analyses, molecular determinants and functional alterations of neuronal and astrocytic energy metabolism in FTD3 were characterized. Our findings implicate dysregulation of glutamate-glutamine homeostasis in FTD3 pathogenesis. In addition, FTD3 neurons recapitulate glucose hypometabolism observed in FTD patient brains. The impaired mitochondria function found here is concordant with disturbed TCA cycle activity and decreased glycolysis in FTD3 neurons. FTD3 neuronal glutamine hypermetabolism is associated with up-regulation of PAG expression and, possibly, ROS production. Distinct compartments of glutamate metabolism can be suggested for the FTD3 neurons. Endogenous glutamate generated from glutamine via PAG may enter the TCA cycle via AAT (left side of neuron) while exogenous glutamate taken up from the extracellular space may be incorporated into the TCA cycle via GDH (right side of the neuron) FTD3 astrocytic glutamate uptake is upregulated whilst glutamate metabolism is largely maintained. Finally, pharmacological reversal of glutamate hypometabolism manifesting from decreased GDH expression should be explored as a novel therapeutic intervention for treating FTD3. 
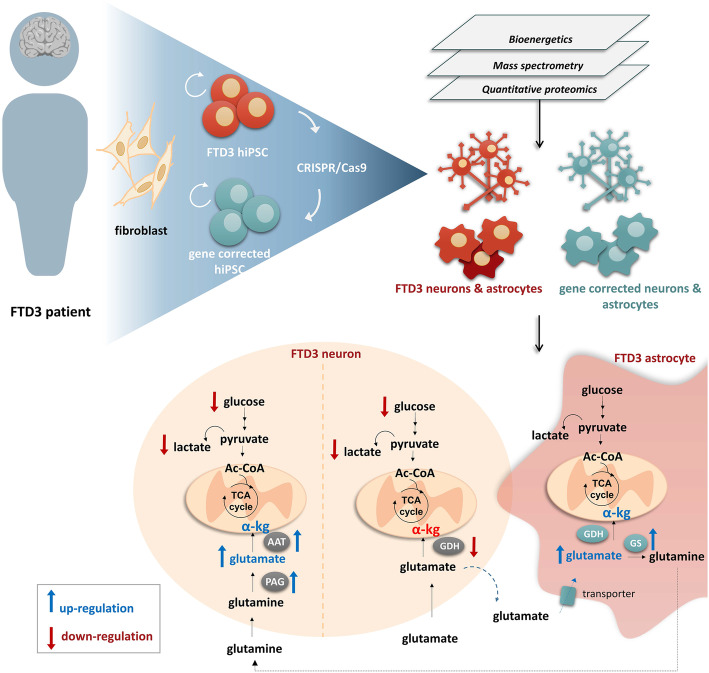

## Introduction

Frontotemporal dementia (FTD) is a leading type of early-onset dementia and involves progressive brain atrophy largely affecting the frontal and temporal lobes of the brain [1, 2]. While FTD has conventionally been regarded as a syndrome characterized by behavioural and cognitive perturbations, a critical involvement of cerebral metabolic alterations in FTD pathology is now being increasingly recognized [3–5].

Normal brain function requires a large and continuous supply of oxygen and glucose for energy production used mainly to satisfy the high demand of electrical activity and synaptic functions in neurons [6]. Moreover, strict regulation of glutamate and glutamine metabolism is vital both for energy homeostasis and for excitatory neurotransmission [7]. Several neurodegenerative disorders are associated with critical metabolic impairments including diminished glucose uptake and utilization, as well as hampered mitochondrial activity, with a subsequent decrease in ATP production even preceding the presentation of major pathophysiological phenotypes and symptoms [3, 8–11]. Hence, hypometabolism of glucose detected by positron emission tomography has been utilized as a reliable biomarker of disease progression in both patients and animal models of neurodegeneration [8]. Nonetheless, the changes in the metabolic landscape at a cellular level in such neurodegenerative disorders remain poorly characterized.

Most FTD cases are sporadic, yet approximately 20–30% is genetically linked. Mutations in specific genes associated with such familial FTD cases have been identified [2]. Amongst these, the *CHMP2B* gene encoding charged multivesicular body protein 2B located on chromosome 3 and causative for FTD3 is of particular interest [12]. CHMP2B is a component of the “Endosomal Sorting Complex Required for Transport III” (ESCRT-III) involved in endo-lysosomal trafficking [13]. Thus, in FTD3, a dominant gain-of-function mutation of CHMP2B affects endo-lysosomal-mediated functions such as recycling or degradation of cell surface receptors. Even though mutation carriers can be easily identified, there is currently no therapy to cure, halt or even decelerate disease progression. This is in part reflective of a paucity of human disease models with which to dissect the mechanisms underlying FTD3 pathogenesis. In an attempt to meet such a need, we have recently developed a human disease model using human induced pluripotent stem cells (hiPSCs) from patients carrying mutations in *CHMP2B* and isogenic controls generated via the CRISPR/Cas9 system with subsequent differentiation into functional neurons expressing typical markers for the frontal and temporal lobe [14]. Pathologies observed in patients and animal models such as dysregulation of the endosome-like structure functionality [15, 16] were validated in our model. Furthermore, our results revealed novel disease-relevant phenotypes including abnormal mitochondrial morphology and function [14]. We therefore sought to gain a more comprehensive understanding of the molecular pathogenesis in FTD3 and to identify key cellular changes in neuronal and glial energy metabolism. Using an integrative approach comprising quantitative proteomics, metabolic mapping via stable isotope ^13^C-labeled energy substrates and gas chromatography coupled to mass spectrometry, as well as live-cell bioenergetics analysis, we have identified a constellation of metabolic changes that lead us to conclusively identify two major enzymes linking neurotransmitter and energy metabolism as crucial players in FTD3 pathology.

## Results

### Quantitative profiling reveals differential expression of key proteins involved in neuronal metabolism in FTD3

**Fig. 1:**
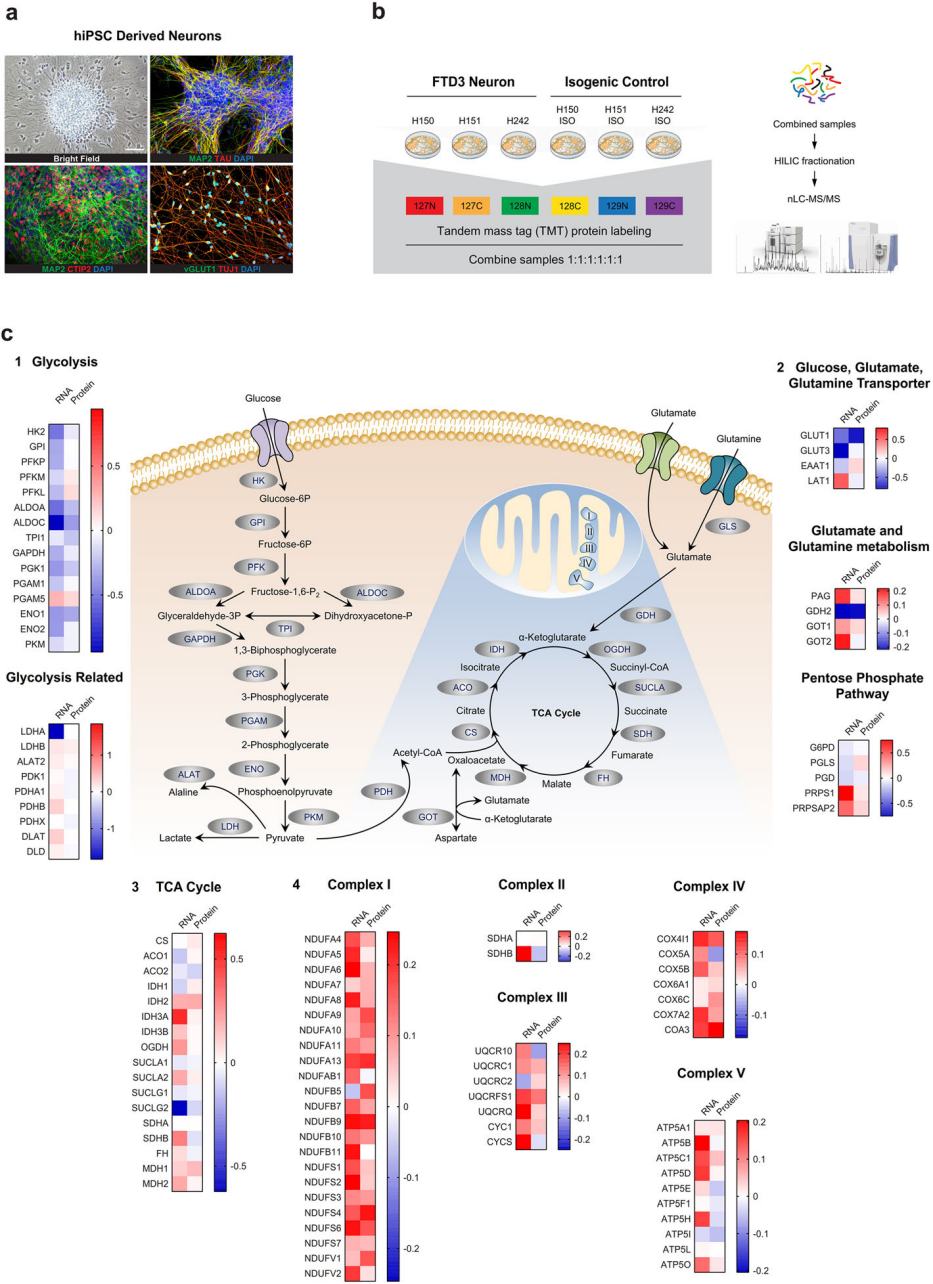
Genes encoding proteins regulating energy metabolism are differentially expressed in FTD3 versus control neurons derived from patient-specific iPSCs. **a** Immunocytochemistry of hiPSC-derived neurons for MAP 2, TAU, CTIP2, vGLUT1 and TUJ1. Scale bar, 50 μm for all. **b** Schematic of tandem mass tag (TMT) protein labelling of hiPSC-derived neurons for mass spectrometry-based quantitative analyses. **c** Key metabolism-relevant genes and metabolites involved in energy metabolic pathways including glycolysis and oxidative metabolism. Heatmaps for gene expression changes determined at both mRNA and protein levels, including genes encoding proteins responsible for glycolysis, the tricarboxylic acid (TCA) cycle, the mitochondrial electron transport chain and glucose and amino acid transportation. mRNA expression data were extracted from previously-published RNA-seq data. Fold-changes are represented by Log2 ratio for FTD3 neurons versus isogenic controls

Neurons derived from FTD3 iPSC (Fig. 1a) exhibit cristae-deficient mitochondria with, consequent dysfunctional respiratory capacity, which are rescued in CRISPR/Cas9 isogenic controls with repaired mutations [14]. In order to more comprehensively dissect this metabolic phenotype and gain insight into FTD3 pathogenesis, we set out to investigate changes in expression of key proteins involved in mitochondrial function and neuronal energy metabolism in FTD3 versus repaired control iPSCs via quantitative mass spectrometry (Fig. 1b). Systematic transcriptomic and proteomic analyses were performed (Fig. S1) and revealed 104 metabolically-related differentially-expressed proteins involved in glycolysis, tricarboxylic acid (TCA) cycle and the electron transport chain (Fig. 1c). All of the identified targets were transcriptionally dysregulated in our previously published RNA sequencing assay [14] and the majority of them shared the same trend both at transcriptional and translational levels. Amongst these significantly differentially-expressed proteins were key metabolic enzymes such as hexokinase (HK2) and enolase (ENO1), both important mediators of the glycolytic pathway (Fig. 1 c_1_), isocitrate dehydrogenase (IDH2) and malate dehydrogenase (MDH1), both involved in the TCA cycle, glutamate dehydrogenase (GDH2-human isoform), an enzyme essential for the interconversion of amino acids and carbohydrates (Fig. 1 c_2,_ c_3_), and several NADH dehydrogenases including NDUFA, NDUFB, NDUFS and NDUFV, which comprise part of complex I of the mitochondrial respiratory chain (Fig. 1 c_4_). Furthermore, the glucose transporter GLUT1 was significantly down-regulated as shown in Fig. 1 c_2_. Meanwhile, although gene expression for the glutamine transporter (L-type amino acid transporter 1, LAT1) which is involved in amino acid homeostasis was significantly up-regulated, no significant change in protein expression was detected (Fig. 1 c_2_). Finally, the expression of phosphate-activated glutaminase (PAG) was increased both at the transcriptional and translational levels (Fig. 1 c_2_). Collectively, our findings reveal a differential protein expression profile underlying metabolic dysregulation in FTD3 neurons. We therefore sought to assess the functional consequences in our human FTD3 model.

### FTD3 neurons exhibit decreased glycolytic activity and increased mitochondrial TCA turnover

**Fig. 2:**
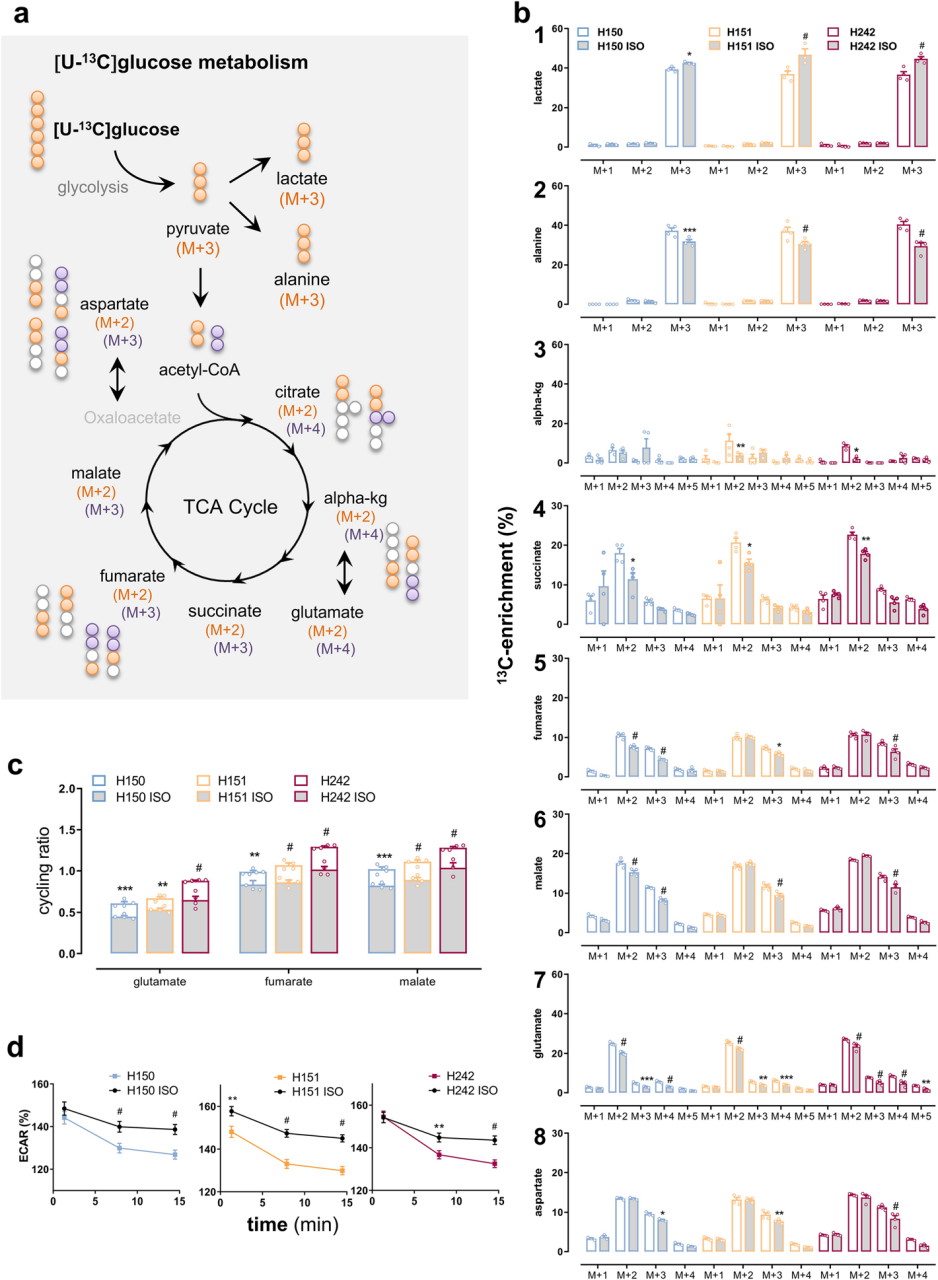
FTD3 neurons display glucose hypometabolism contrasting with increased TCA cycle activity. **a** Schematic representation of the main labelling patterns obtained after incubation with [U-13C] glucose and ^13^C-enrichment depicted as percentage labelling in metabolites from neuronal glucose metabolism. **b** Cultures were incubated for 90 min with [U-^13^C] glucose (2.5 mM) then cell extracts were collected and analysed using GC-MS for determination of the percentage distribution of ^13^C-labelled metabolites in CRISPR/Cas9-generated isogenic controls (grey bars) and the respective FTD3 patient cell lines (H150, H151 and H242; white bars). [U-^13^C] glucose is metabolized to [U-^13^C] pyruvate during glycolysis which can either be converted to [U-^13^C] alanine (alanine M + 3) or be rapidly transformed into [U-^13^C] lactate (lactate M + 3). In addition, pyruvate can be further metabolized in the TCA cycle, entering as [1,2-^13^C]Acetyl-Coenzyme A (Ac-CoA [1,2-^13^C]) which reacts with unlabelled oxaloacetate. Hence, double-labelled TCA cycle metabolites are produced from the first turn metabolism of [1,2-^13^C]Ac-CoA in the TCA cycle [18] while a second turn of the TCA cycle yields M + 4 and M + 3 labelled metabolites. Subsequent turns will give rise to complex labelling patterns and labelling in further isotopologues (M + X). Decreased labelling (%) in lactate M + 3 indicates reduced glycolytic activity while increased labelling in the TCA cycle metabolites and amino acids indicates increased TCA cycle activity, directly evaluated by calculating the cycling ratios shown in **c** as the ratio of % labelling in isotopologue (M + X) from every other TCA cycle turns divided by M + 2% labelling obtained from a first turn of the TCA cycle (see *Methods*). These showed increased TCA cycle turnover (staked white bars) in the FTD3 neurons. Data are presented as labelling (%) of M + X, where M is the mass of the unlabeled metabolite and X is the number of ^13^C-labeled carbon atoms. **d** Extracellular acidification rate as an indicator of glycolytic activity was measured in real using the XF^e^96 Extracellular Flux Analyzer (Seahorse Biosciences-Agilent Technologies) with glucose as substrate in isogenic controls (black lines) and FTD3 patient cell lines (color lines). ECAR is denoted as percentage of the respective non-glycolytic medium acidification values (last point taken at the end of the assay). The decrease in ECAR demonstrates diminished lactate release from hampered glycolytic activity. Results are means ± S.E.M. obtained from three different patient cell lines indicated by small circles from at least three different culture preparations of hiPSC-derived neurons. *P < 0.05 or **P < 0.001, ***P < 0.0005, ^#^ *P* < 0.0001, two-way ANOVA correcting for multiple comparisons was employed

It is widely accepted that glucose hypometabolism is a pathological hallmark of several neurodegenerative disorders including FTD [9, 17]. With the aim of elucidating whether such glucose hypometabolism is present in FTD3 hiPSC-derived neurons and in order to further investigate the consequences at the cellular level, FTD3 hiPSC-derived neurons and hiPSC-derived neurons from their isogenic controls were incubated with ^13^C-labelled substrates followed by subsequent mapping of the metabolic pathways by mass spectrometry. Intracellular labelling was obtained after incubation with uniformly ^13^C-labelled glucose ([U-^13^C] glucose, 2.5 mM) (Fig. 2a). Cultured neurons metabolize [U-^13^C] glucose to [U-^13^C] pyruvate during glycolysis which in turn can either be converted to [U-^13^C] alanine (alanine M + 3) or be rapidly transformed into [U-^13^C] lactate (lactate M + 3), which can be detected reflecting the overall glycolytic capacity [18, 19]. In addition, pyruvate can be further metabolized in the TCA cycle, entering as [1,2-^13^C]Acetyl-Coenzyme A (Ac-CoA [1,2-^13^C]) which reacts with unlabelled oxaloacetate and, hence, double-labelled TCA cycle metabolites are produced. The labelled neuroactive amino acids [4,5-^13^C] glutamate (M + 2) and [1,2-^13^C] aspartate (M + 2) can be formed from the TCA cycle intermediates α[4,5-^13^C] ketoglutarate (α-kg M + 2) and [1,2-^13^C] oxaloacetate by the action of GDH (GDH1 and 2 in humans) and aspartate aminotransferase (GOT), respectively, and exit the TCA cycle. Thus, the observed double-labelled metabolites and amino acids are obtained from the first turn metabolism of [1,2-^13^C]Ac-CoA in the TCA cycle [18]. Figure 2b shows the relative abundance of isotopologues of detected metabolites derived from glycolysis and the TCA cycle in FTD3 neurons (patient H150, H151 and H242) and the respective isogenic controls (H150 ISO, H151 ISO and H242 ISO). Our results show that the labelling percentage in lactate M + 3 (Fig. 2 b_1_) was reduced ~ 15% in FTD3 neurons from each patient compared with the respective isogenic controls (44.5 ± 1.4% labelling for FTD3 (pooled) versus 38 ± 1.4% for isogenic controls (pooled); *p = 0.01*). In contrast, the labelling in alanine M + 3 (Fig. 2 a_2_) and in the TCA intermediates arising from a first turn of the TCA cycle, α-kg M + 2 (Fig. 2 a_3_), succinate (Fig 2 a_4_), as well as the labelling in glutamate M + 2 (Fig. 2 a_7_) was consistently increased in FTD3 neurons from all patients compared to the isogenic control (with the exception of α-kg M + 2 in patient H150, which was not different form the respective H150 isogenic control). In a second turn of the TCA cycle, [1,2-^13^C]Ac-CoA reacts with oxaloacetate M + 2 formed in the first turn, resulting in the production of M + 3 and M + 4 labelled metabolites. Interestingly, FTD3 neurons from the three patients showed increased labelling compared to their isogenic controls in metabolites and amino acids obtained from a second turn of the TCA cycle including glutamate M + 4, succinate; fumarate, malate and aspartate M + 3 (Fig 2 b_4–7_).

The accumulation of ^13^C-labeling in metabolites obtained after [U-^13^C] glucose metabolism may indicate increased TCA cycle turnover. Furthermore, the quantitative proteomic data revealed modestly upregulated expression of TCA cycle enzymes (Fig. 1 c_3_). We therefore calculated a TCA cycling ratio in order to obtain a measure of the overall mitochondrial TCA cycle activity. These ratios were calculated based on labelling of glutamate, fumarate and malate according to the following formula: ([M + 1] + [M + 3] + … [M + n]/[M + 2]), i.e. the labelling originating from the second or more turns of TCA cycle metabolism divided by the labelling occurring after the first turn [20]. As suspected, the cycling ratios for all specific TCA metabolites and amino acids in FTD3 neurons from each patient were significantly increased by ~ 25% compared to their isogenic controls (Fig. 2c). The labelling patterns (%) are obtained after 90 min of incubation where a steady state has not been reached and dynamic labelling is obtained instead. We measured the total amounts of the TCA cycle metabolites succinate and malate, as well as the absolute amounts of the M + 2 isotopologues to rule out the possibility that the increase labelling (%) in subsequent turns of the TCA cycle is due to smaller pools of intermediates that would be labelled faster in the FTD3 neurons compared to the isogenic controls (Additionale file 2). We found an increased total amount of succinate and malate, as well as increased amount of double labelled (M + 2) intermediates in neurons from patients H150,H151 and H242 compared to their respective isogenic controls (Additional file 1). Moreover, in FTD3 neurons we detected a reduced coupling efficiency (in %) between oxygen consumption and ATP production during mitochondrial respiration, compared to isogenic controls (FTD3 neurons 70.9 ± 2.0% vs. isogenic control 77.9 ± 1.1%, *p* = 0.0044 unpaired multiple *t* test). This indicates uncoupling of mitochondria in FTD3 neurons.

Monitoring lactate release produced after glucose breakdown using the Seahorse instrument comprises a parallel approach to assess glycolytic activity [21]. Using this method, we analysed the glycolytic capacity of FTD3 neurons and isogenic controls in real-time. The extracellular acidification rate (ECAR) obtained as a surrogate measure of glycolysis was similarly decreased in FTD3 neurons from all patient cell line compared to their respective isogenic control (Fig. 2d). Taken together, the reduced ^13^C-enrichment in lactate and diminished ECAR reveal reduced glycolytic activity in FTD3 neurons.

### Amino acid homeostasis is perturbed in FTD3 neurons owing to compromised expression and function of key enzymes in glutamine and glutamate metabolism

**Fig. 3:**
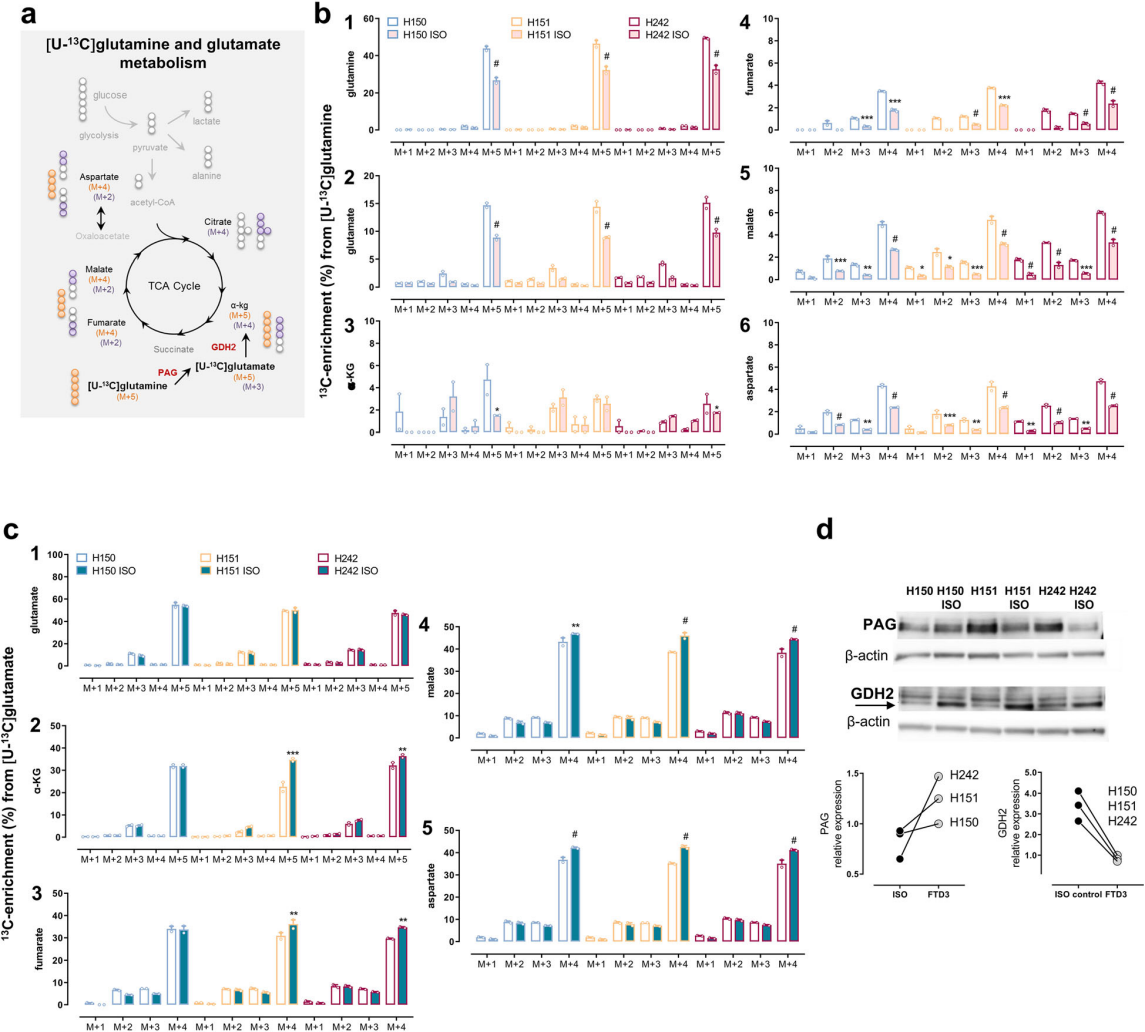
Dysregulation of glutamine and glutamate metabolism mirror each other in FTD3 neurons. **a** Scheme depicting the main labelling patterns from neuronal [U-^13^C] glutamine and [U-^13^C] glutamate metabolism and ^13^C-enrichment as percentage labelling in metabolites. Cultures were incubated for 90 min with b [U-^13^C] glutamine (0.5 mM), or c [U-^13^C] glutamate (0.25 mM) in the presence of unlabelled glucose (2.5 mM) then cell extracts were collected and analysed using GC-MS for determination of the percentage distribution of ^13^C-labelled metabolites in isogenic controls (pink bars/green bars) and the respective FTD3 patient cell lines incubated with ^13^C-labelled glutamine or glutamate (white bars). **b** The increased labelling in the TCA cycle metabolites and amino acids after [U-^13^C] glutamine incubation indicates increased metabolism of this amino acid. **c** In contrast, unmodified labelling in glutamate M + 5 and increased labelling in direct metabolites from [U-^13^C] glutamate indicates intact glutamate transport but decreased glutamate metabolism. **d** Protein expression assayed for by Western blot analyses of the two main enzymes involved in glutamine and glutamate degradation, namely PAG and GDH, respectively. Expression of PAG protein was upregulated while that of GDH was substantially reduced in FTD3 neurons from the three different patient cell lines (H150, H151 and H242). β-Actin was used as loading control. Results represent means ± S.E.M. obtained from three different patient cell lines indicated by small circles from three different culture preparations of hiPSC-derived neurons. *P < 0.05 or **P < 0.001, ***P < 0.0005, ^#^ *P* < 0.0001, two-way ANOVA correcting for multiple comparisons was employed

Glutamate is the major excitatory neurotransmitter in the brain and, as such, perturbations in glutamate content and signalling have been identified as prominent features of several neurological disorders [22, 23]. In addition, both glutamate and glutamine are of utmost importance as energy substrates and neurotransmitter precursors with glutamine being the direct precursor of glutamate [19, 24–26]. Based on the observed significant changes in expression of enzymes (Fig. 1 c_2_) involved in amino acid metabolism, we next addressed the functional impact of this on glutamate and glutamine homeostasis in FTD3 neurons and isogenic controls. FTD3 hiPSC-derived neurons and their isogenic controls were incubated in medium containing [U-^13^C] glutamine (500 μM) or [U-^13^C] glutamate (250 μM) in the presence of unlabelled glucose. Initially, [U-^13^C] glutamate is formed from [U-^13^C] glutamine in a deamidation reaction catalysed by phosphate-activated glutaminase (PAG) and may subsequently enter the TCA cycle via α-[U-^13^C] kg (Fig. 3a). The labelling (in %) of amino acids and TCA cycle intermediates produced during the direct metabolism of [U-^13^C] glutamine or [U-^13^C] glutamate and after condensation of Ac-CoA, i.e. the first turn into TCA cycle was measured in the hiPSC-derived neurons (Fig. 3b,c). Strikingly, the intracellular ^13^C-labelling in all metabolites and amino acids obtained from the direct metabolism of [U-^13^C] glutamine and after a first turn of the TCA cycle was increased in FTD3 neurons compared to isogenic controls (Fig. 3 b_1–6_).

Exogenous [U-^13^C] glutamate added to the incubation medium follows a similar metabolic fate as the labelled glutamate formed from [U-^13^C] glutamine (Fig. 3a). Direct metabolism from exogenous [U-^13^C] glutamate can give rise to compartmentalized pools of metabolites and diverse labelling patterns. The intracellular labelling in glutamate M + 5 was unchanged in FTD3 neurons from patient H150, H151 and H242 compared to the respective isogenic controls (50.1 ± 2.2% for FTD3 (pooled) versus 50.7 ± 2.1% for isogenic control (pooled); *p = 0.1*; Fig 3 c_1_). However, the labelling (M + 4) in metabolites produced after direct metabolism of [U-^13^C] glutamate such as fumarate, malate and aspartate was significantly decreased in FTD3 neurons compared to isogenic controls (with the exception of fumarate and α-kg M + 4 in H150, Fig 3 c_2–5_), in contrast to the first turn TCA cycle in which metabolites were unmodified in FTD3 neurons compared with isogenic controls.

The divergent labelling patterns (from controls) defined above in FTD3 neurons reflect dysregulated enzymatic function that might potentially be associated with altered expression of enzymes involved in glutamine and glutamate metabolic pathways. PAG is considered to be the most significant and rate-limiting glutamine-metabolizing enzyme responsible for the synthesis of glutamate [27]. The labelling patterns obtained from [U-^13^C] glutamine metabolism in FTD3 neurons revealed increased labelling of such metabolites consistent with an increase in PAG activity. In order to conclusively link the increased glutamine metabolism to PAG upregulation, we further evaluated its protein expression in neurons. As anticipated, we consistently detected increased expression of PAG in FTD3 neurons in all patient cell lines compared with their respective isogenic controls (Fig. 3d), mirroring the upregulated expression at RNA and protein levels (Fig. 1).

Nevertheless, increased PAG expression cannot be held accountable for the decreased labelling in metabolites obtained after direct metabolism of [U-^13^C]glutamate. Another key enzyme in brain glutamate homeostasis is glutamate dehydrogenase (GDH) as it links carbohydrate and amino acid metabolism by metabolizing glutamate into α-kg being a constituent of the TCA cycle. Of the known GDH isoforms, GDH1 is known to be expressed ubiquitously in the brain but GDH2 is the isoform only expressed in humans. We next analysed the expression of GDH2 in FTD3 neurons derived from the different patients and their respective isogenic controls. The expression of GDH2 was profoundly decreased in FTD3 neurons compared with their isogenic controls (Fig. 3d), reflecting the decreased ^13^C-enrichment of metabolites obtained from [U-^13^C]glutamate.

### Intracellular glutamine content is significantly decreased in FTD3 neurons

Intracellular glutamine and glutamate contents (nmol/mg protein) were determined by HPLC to further corroborate the observed functional dysregulation in the metabolism of these amino acids. Total amounts of glutamine and glutamate were sorted according to the ^13^C-labelled substrate used during the incubations (Table 1). Interestingly, glutamine content was reduced by approximately 50% in FTD3 neurons compared with isogenic controls under all incubation conditions. This result is in accordance with the overexpression of PAG observed in both proteomic and Western blot analyses. In contrast, no significant changes were detected in glutamate concentration in FTD3 neurons and isogenic controls under the same conditions.

**Table 1:** Glutamine content is significantly diminished in FTD3 neurons derived from hiPSCs

*substrates present during incubation*	***amino acid***	***isogenic control neurons***	***FTD 3 neurons***	***P value***	
*amounts (nmol/mg protein)*					
***[U-*** ^***13***^***C]glucose***	Glutamine	**12 ± 1.7**	**6 ± 1.3**	***0.005***	^a^
	Glutamate	61 ± 4.3	51 ± 2.9	*0.14*	
***[U-*** ^***13***^***C]glutamine*** *+ glucose*	Glutamine	**18 ± 1.3**	**13 ± 1.5**	***0.008***	^a^
	Glutamate	68 ± 5.1	59 ± 4.0	*0.26*	
***[U-*** ^***13***^***C]glutamate*** *+ glucose*	Glutamine	**14 ± 1.5**	**7 ± 0.7**	***0.024***	^a^
	Glutamate	185 ± 18.8	185 ± 18.8	*1.00*	

Cellular content of amino acids in cultured neurons derived from hiPSCs. Cultured neurons were incubated for 90 min with 2.5 mM [U-13C] glucose or 0.25 mM [U-13C] glutamate or 0.5 mM [U-13C] glutamine in the presence of 2.5 mM unlabeled glucose. Cell extracts were analyzed by HPLC for determination of amino acid content. Results are mean ± SEM from 3 individuals (6 different cultures of hiPSCs-derived neurons/condition). Unpaired multiple t test followed by Bonferroni-Dunn post hoc test was used for statistical evaluations. *P* values were calculated comparing FTD3 neurons agains isogenic controls, ^a^ denotes statistical significance

### Glutamate uptake and metabolism is increased in FTD3 astrocytes

**Fig. 4:**
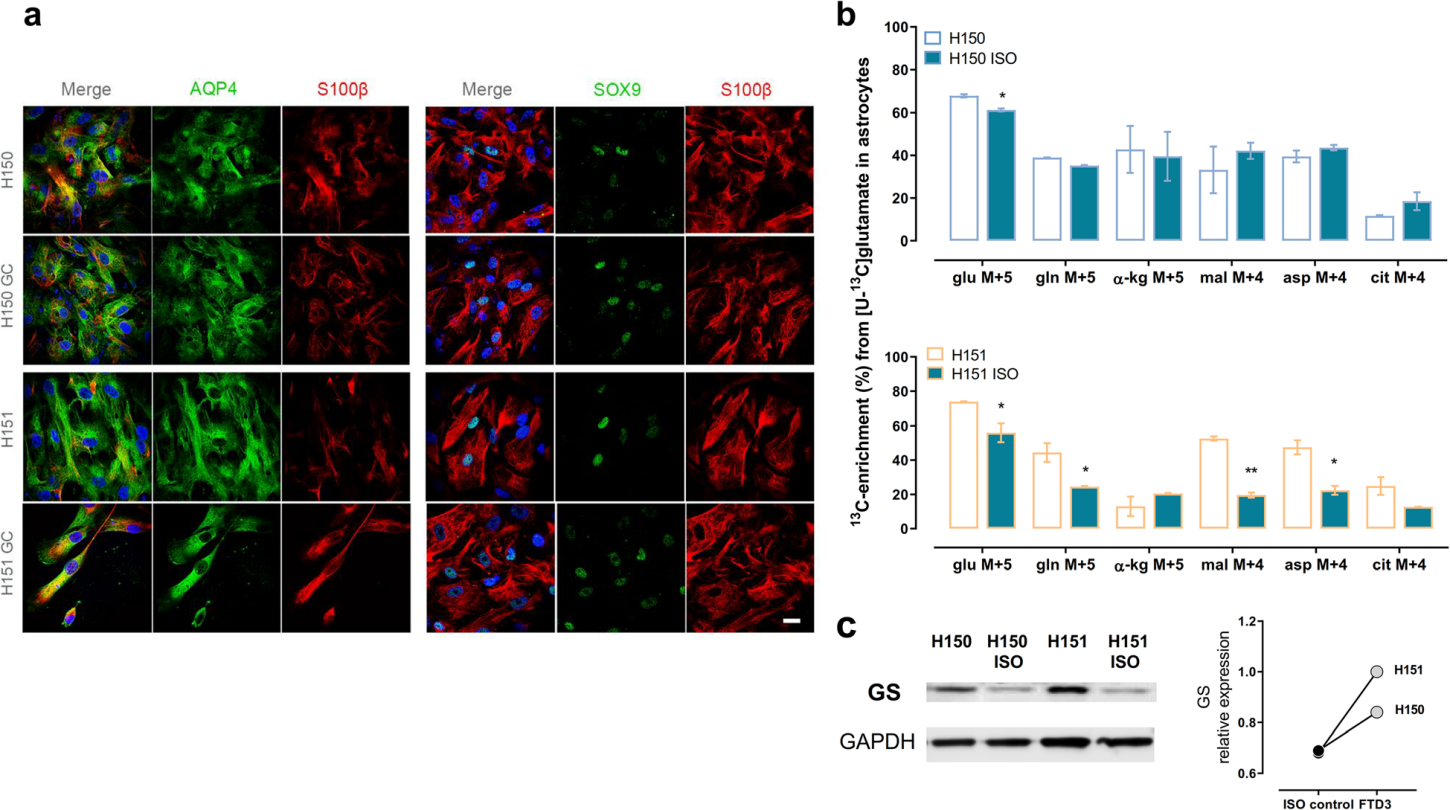
Increased glutamate uptake and metabolism in FTD3 astrocytes. **a** Representative immunocytochemistry images of astrocytes from FTD3 patient cell lines (H150 and H151) and their respective isogenic control cell lines at 10 weeks of maturation. All cell lines express astrocyte markers Aquaporin-4 (AQP4), S100beta (S100β) and SOX9. Scale bar: 25 μm. **b** Percentage distribution of ^13^C-labelled metabolites obtained from direct metabolism [U-^13^C] glutamate in FTD3 astrocytes from the patient cell lines (white bars) and their respective isogenic controls (green bars). Astrocytes in culture were incubated for 90 min with [U-^13^C] glutamate (0.25 mM) in the presence of unlabelled glucose (2.5 mM): astrocyte extracts were subsequently collected and analysed using GC-MS for determination of ^13^C-enrichment. The increased labelling in the intracellular glutamate and other TCA cycle metabolites and amino acids after [U-^13^C] glutamate incubation indicates increased uptake and metabolism of this amino acid. Results represent means ± S.E.M. obtained from three different patient cell lines from three different culture preparations of hiPSC-derived neurons. *P < 0.05 or **P < 0.001; two-way ANOVA correcting for multiple comparisons was employed. **c** Representative Western blot analyses of protein expression of glutamine synthetase (GS). Glyceraldehyde 3-phosphate dehydrogenase (GAPDH) was used as loading control. Expression of GS protein was found to be upregulated in FTD3 neurons from the two different patient cell lines (H150 and H151)

The glutamate-glutamine cycle involves the shuttling of glutamate from neurons and glutamine from astrocytes, both essential for sustaining neuronal activity [28]. Glutamate metabolism in astrocytes provides a mechanism for tight coupling between synaptic activity and energy metabolism. In order to investigate metabolic defects in astrocytes and to indirectly assess whether the metabolic coupling between neurons and glia might be affected in FTD3, two FTD3 patient cell lines and isogenic controls were differentiated into astrocytes. Their identity was validated via immunocytochemistry (ICC) using the astrocyte-specific markers SRY-Box Transcription Factor 9 (SOX9), S100 Calcium Binding Protein B (S100B) and Aquaporin-4 (AQP4) (Fig. 4a) at 10 weeks of maturation. No obvious differences were detected between FTD3 astrocyte cultures and their respective isogenic controls. 10-week-old astrocytes were incubated with exogenous [U-^13^C] glutamate and the level of ^13^C enrichment in selected metabolites was measured. Glutamate taken up by astrocytes can either be amidated to glutamine in a reaction catalysed by the enzyme glutamine synthase (GS), almost exclusively found in astrocytes, or can be converted to α-kg by aspartate amino transferase or GDH [29]. Our results revealed increased intracellular labelling in glutamate M + 5 in FTD3 astrocytes that was reversed in the respective isogenic controls (Fig. 4b). This increase may suggest either increased glutamate uptake or decreased metabolism. The latter hypothesis was rejected as no differences were found between the labelling patterns obtained from direct metabolism of [U-^13^C] glutamate in FTD3 astrocytes versus isogenic controls for the patient cell line H150. Furthermore, a trend towards increased ^13^C-enrichment from metabolism of [U-^13^C] glutamate in FTD3 astrocytes was observed in the H151 patient cell line (Fig. 4b**).** This trend became significant for some metabolites (malate and aspartate), suggesting increased metabolism of glutamate in FTD3 H151 astrocytes compared to the respective isogenic control. Likewise, a significant increase of ^13^C-enrichment in glutamine M + 5 was observed in the H151 patient cell line whilst the H150 patient cell line showed a trend towards increased labelling. These observations were consistent with increased expression of glutamine synthetase (GS), the rate limiting enzyme converting glutamate to glutamine, in the FTD3 astrocytes compared to the isogenic controls (Fig. 4c).

## Discussion

Here, we provide molecular and functional evidence of major changes in neuronal energy metabolism in *CHMP2B* mediated Frontotemporal Dementia (FTD3) using patient-derived iPSC disease models. Furthermore, we uncover dysregulation of the metabolic enzymes phosphate-activated glutaminase (PAG) and glutamate dehydrogenase human isoform 2 (GDH2), crucial regulators in brain glutamate-glutamine homeostasis, in FTD3 pathogenesis. Our proteome analysis demonstrates that the expression of key proteins involved in energy metabolic pathways including glycolysis and oxidative metabolism is dysregulated in FTD3 neurons, which prompted us to further investigate the functional metabolic consequences of utilizing distinct energy substrates via mass spectrometry mapping. We validated that the FTD3 neurons in our model exhibited reduced glucose metabolism as demonstrated by down-regulated expression of glycolytic enzymes, as well as diminished ^13^C-enrichment in lactate produced after metabolism of [U-^13^C] glucose and decreased lactate release. Particularly, we observed decreased expression of enolase (ENO1 and 2) and hexokinase (HK2) which has been suggested to be an important mediator of glycolysis in states of compromised energy supply [30] . This is consistent with glucose hypometabolism, a well-recognized, stereotypical imaging biomarker in several neurodegenerative disorders including FTD and AD [3, 8, 9, 11, 31]. Moreover, we identified a significant decrease in glucose transporter expression, strongly suggesting reduced glucose uptake by neurons which most likely contribute to the observed decrease in metabolism. As a result of hampered glucose uptake and metabolism, compensatory mechanisms to fulfil the neuronal energetic demand for ATP may be triggered. Such mechanisms include increased TCA cycling activity and hence augmented ETC and oxidative phosphorylation, as well as increased oxidative metabolism of alternative substrates e.g. glutamine as further discussed below.

ATP and pyruvate are the principal end-products resulting from glucose breakdown during glycolysis. Pyruvate can be converted into Ac-CoA in mitochondria and incorporated into the TCA cycle for oxidative metabolism and further energy production. Our results show an increased ^13^C-incorporation in TCA cycle metabolites in FTD3 neurons after incubation with [U-^13^C] glucose and a consistent increase in the cycling ratios calculated therein. The cycling ratio provides a comparable measure of TCA cycle activity between cell types or different treatments [20, 32]. Based on this, our finding of increased cycling ratios in all selected metabolites suggests increased TCA cycle turnover. This was supported by an increased concentration of the TCA metabolites succinate and malate in the FTD3 neurons, as well as an increased amount of double labeled (M + 2) metabolites resulting from a first turn of the TCA cycle. Together, these findings suggest that the increased labelling in TCA metabolites observed in the FTD3 neurons compared to the isogenic controls is linked to increased TCA cycle activity and is not due to a smaller metabolite pool that would be more rapidly labelled in the FTD3 neurons. Using the Seahorse instrument in conjunction with our hiPSC-neuronal model of FTD3, we recently identified decreased respiratory activity in mitochondria of FTD3 neurons compared to respective isogenic controls [14]. Coupling efficiency represents the proportion of mitochondrial oxygen consumption used to produce ATP [21]. We observed decreased coupling efficiency in mitochondria from FTD3 neurons, which suggests that basal oxygen consumption might be uncoupled from ATP synthesis. This could explain the observed increase in TCA cycle activity. This may also support a substantial and initial need of glutamine oxidation in order to compensate for reduced glucose metabolism. Additionally, this may also support an uncoupling induced increase in NADH reduction and oxidation. Thus, it may be speculated that defective mitochondria in FTD3 neurons cannot sustain the cellular energy demand and, therefore, the turnover of metabolites in the TCA cycle is increased in an attempt to cope with the limited supply.

Given the high-energy demand and limited regenerative capacity of neurons, impaired functioning of mitochondria can be deleterious to cell survival. In accordance with this, there is ample evidence of impaired mitochondrial function and oxidative damage causing neurodegeneration via reactive oxygen species (ROS) [33, 34]. Another remarkable finding of the present study is that the RNA and protein expression levels of several subunits of mitochondrial complexes, particularly complex I, are increased in FTD3 neurons. The components of the electron transport chain such as complexes I, II and III are indeed an important source of ROS in mitochondria. Consistently, we previously showed that in addition to the morphological and functional deficits in their mitochondria, FTD3 neurons exhibit increased oxidative stress compared with isogenic controls [14]. It is therefore highly plausible that the increased expression of electron transport complexes in combination with defective mitochondrial respiration contributes to the prior-reported increase in oxidative stress. The possible mechanism underlying the increased oxidative stress might be mediated by complex I which is not only overexpressed in FTD3 neurons but also produces large amounts of superoxide when the matrix NADH/NAD^+^ ratio is elevated as a result of increased TCA cycle activity (as also seen in diseased neurons).

Glutamine plays key roles in several metabolic pathways as well as in neuronal signalling so its homeostasis is therefore tightly regulated [10, 19, 35]. Considering that mitochondria represent a major site of glutamine metabolism, this amino acid is critically involved in oxidative stress and mitochondrial dysfunction. In the brain, glutamine is synthesized only by astrocytes and transported to neurons where it acts as precursor of the neurotransmitter glutamate as well as an energy substrate. The main degradation pathway for glutamine occurs in mitochondria and is governed by the enzyme phosphate-activated glutaminase (PAG encoded by the gene GLS), which catalyses glutamine deamination into glutamate [36]. Thus, PAG is a mitochondrial enzyme critical for cellular metabolism. Our quantitative proteomic data revealed increased expression of PAG in FTD3 neurons, which was later confirmed by Western blot. Interestingly, alterations in PAG levels have been associated with Alzheimer’s disease, for instance, increased levels of PAG in the prefrontal cortex of Alzheimer’s disease patients have been reported [37]. To the best of our knowledge our report is the first to show alterations in PAG expression level and activity in FTD. Consistently, we detected increased ^13^C-incorporation in glutamate and in metabolites obtained after incubation with uniformly ^13^C-labelled glutamine, indicating increased metabolism of glutamine in FTD3 neurons compared with isogenic controls. Whether the increased glutamine metabolism contributes to oxidative stress and therefore to the disease phenotype cannot be directly supported by the present data and is the focus of future investigations. However, considering that the hiPSC-derived neurons are cultured in the presence of a higher concentration of glutamine (2 mM) than that used during the incubation experiments (0.5 mM), it is plausible that increased glutamine metabolism in FTD3 neurons is a compensatory mechanism to fuel mitochondria and potentially accelerate ROS production. In the brain, de novo synthesis of glutamine is dependent on the conversion of α-ketoglutarate to glutamate. This process removes intermediates from the TCA cycle which will potentially decrease the ability of the cycle to oxidize pyruvate and produce reducing equivalents for ETC and ATP synthesis. Thus, de novo synthesis of glutamine must be matched with anaplerosis, the replenishment of the pool of TCA cycle intermediates. This is mainly mediated by pyruvate carboxylase (PC) [38], an astrocyte specific enzyme that catalyzes the formation of oxaloacetate from pyruvate. Neurons depend on the astrocytes for de novo synthesis of glutamine as they do not express PC or any other mediators of anaplerosis from glucose. Increasing the capacity of the TCA cycle improves the energetic status of the cell and in order for neurons to benefit from anaplerosis, glutamine derived from TCA cycle intermediates are transferred from astrocytes to neurons where they are re-converted to intermediates, thereby increasing the capacity of the TCA cycle to produce energy [19]. Therefore, it can be speculated that in the FTD3 neurons, the increased glutamine metabolism serves an anaplerotic role.

Glutamine is the precursor of glutamate, the most abundant excitatory amino acid in the brain and a crucial energy substrate, thus serving dual functions in the CNS. A fine-tuned glutamate homeostasis is therefore crucial for proper brain function and, as such, perturbations of the excitatory amino acid system have been implicated in neurodegeneration [39]. The hiPSC-derived neuronal cultures studied here have previously been shown to exhibit a glutamatergic phenotype [14], further emphasizing the suitability of this model to study metabolic alterations in FTD3. We next evaluated the major pathways related to glutamate metabolism in FTD3 neurons and isogenic controls by incubating the cultures with [U-^13^C] glutamate in the additional presence of glucose. Mass spectrometry data revealed that the ^13^C-enrichment in all metabolites obtained from [U-^13^C] glutamate was higher than that from [U-^13^C] glutamine, suggesting that exogenous glutamate is more intensely metabolized than exogenous glutamine in both FTD3 and isogenic control neurons. This preference for glutamate as a metabolic substrate has been observed in previous studies of hiPSC-derived neurons from healthy individuals and in mouse glutamatergic neurons [25, 40]. We did not detect differences in the labelling of [U-^13^C] glutamate in FTD3 neurons compared with isogenic controls which suggests that the transport of glutamate into neurons is unaffected. This is supported by the proteomic data showing no significant changes in expression of glutamate transporters between FTD3 and control neurons. Interestingly, our results show a reduced ^13^C-enrichment in metabolites measured after direct metabolism of [U-^13^C] glutamate in FTD3 neurons compared with isogenic controls, demonstrating decreased metabolism of the excitatory neurotransmitter.

GDH catalyzes the interconversion of glutamate and α-ketoglutarate, and is a crucial enzyme for glutamate homeostasis in the brain [41]. GDH links amino acid and carbohydrate metabolism by mediating glutamate oxidation and expanding the TCA cycle capacity through intermediate replenishment [42]. Mammals express the housekeeping isoform GDH1, whereas only humans and primates express an additional isoform, GDH2 [43]. These isoforms significantly differ in enzymatic properties including allosteric regulation and it has been speculated that based on its particular enzymatic characteristics, GDH2 may be important to potentially increase glutamate oxidation during energy-demanding processes [44–46]. Recently, we have shown that human GDH2 expression increases uptake of glutamate and capacity for its oxidative metabolism in the brain of hGDH2-expressing transgenic mice which supports that GDH2 has important consequences for carbon metabolism particularly during metabolic challenges such as augmented workload and hypoglycaemia [47]. Via quantitative proteomics, we detected reduced expression of GDH and we anticipated that decreased glutamate metabolism is a functional consequence of GDH down-regulation. Evaluation of GDH2 protein expression by Western blot confirmed decreased GDH2 expression in every FTD3 cell line compared to its respective isogenic control. Together, these parallel approaches have allowed us to implicate the involvement of a crucial metabolic enzyme, GDH in FTD3 pathogenesis. Strikingly, recent evidence shows that GDH may be a potential therapeutic target in neurodegeneration: increasing its activity might enable it to play a neuroprotective role in energy-depleted conditions [22, 48].

Furthermore, the FTD3 patient-derived neurons were incubated with either [U-^13^C] glutamate or [U-^13^C] glutamine, also allowing for a comparison between the two different substrates. It has previously been shown in glutamatergic neurons that the percentage of labelling in later turns of the TCA cycle compared to direct labelling from [U-^13^C] glutamine in the presence of unlabelled glutamate was higher than that from [U-^13^C] glutamate in the presence of unlabelled glutamine [25]. This suggests that glutamate derived from glutamine is used to a higher extent to sustain the pool of TCA cycle intermediates through the action of GDH, i.e. as an anaplerotic substrate, than exogenous glutamate. Interestingly, our results show that the expression of PAG that converts glutamine to glutamate was significantly increased in the FTD3 neurons. Since the glutamine-derived glutamate originating from PAG activity is thought to be the main substrate for the anaplerotic function of GDH, the increase in PAG expression may be a compensatory response to the decreased level of GDH expression. The anaplerotic function of GDH appears to be critical for the capability of neurons to metabolize exogenous glutamate through the activity of aspartate aminotransferase. Thus, increasing anaplerosis through GDH by producing more glutamine-derived glutamate could rescue the ability of the neuron to metabolize glutamate taken up from the synapse potentially avoiding excitotoxicity by excess glutamate levels in the synapse. Upon glutamatergic neurotransmission, glutamate must be rapidly removed from the synaptic cleft where it is predominantly taken up by astrocytes via high-affinity glutamate transporters, and to a lesser extent, by re-uptake into the presynaptic neuron [49, 50]. In astrocytes, glutamate can either be converted to glutamine by astrocyte-specific GS or entry into the TCA cycle by GDH or aminotransferases. Glutamine is subsequently transported to neurons to be converted back to glutamate to either support energy metabolism in neurons or replenish the pool of neurotransmitter glutamate. Thus, modulation of extracellular glutamate levels is crucial for maintenance of brain function and abnormalities in this process have been implicated in several neurodegenerative diseases [51]. The principal regulator of extracellular glutamate levels in the human brain is the excitatory amino acid transporter 2 (EAAT2). Proper expression and regulation of this transporter is critical for maintaining brain homeostasis. Recently, it was shown that pharmacological interventions to stimulate the uptake of glutamate by astrocytic glutamate transporters (EAAT2/GLT1) prevented excitotoxic neuronal death in a transgenic mouse model of FTD pathology [52]. Interestingly, we found an increase in fully labelled intracellular glutamate after incubation with [U-^13^C] glutamate in the FTD3 patient-derived astrocytes, suggesting increased glutamate uptake compared to the respective isogenic control. In addition, maintained (patient H150) or increased (H151) incorporation of the ^13^C-label in selected metabolites was obtained from direct metabolism of [U-^13^C] glutamate in the FTD3 astrocytes. We can only speculate that the observed elevated glutamate uptake may be an astrocyte-inherent protective mechanism against excitotoxicity. In addition, it can be suggested that the elevated glutamate uptake capacity in the astrocytes might be in response to increased glutamate shuttling form neurons which possibly release the excess glutamate that is not incorporated in the TCA cycle due to hampered GDH levels. Furthermore, we found increased GS expression in the FTD3 astrocytes associated with elevated glutamate to glutamine conversion. It is thus possible to suggest that the increased conversion of glutamate to glutamine in FTD3 astrocytes may be the metabolic mechanism behind the increased glutamine uptake by FTD3 neurons. However, the concerted metabolic functions of FTD3 neurons and astrocytes warrant further investigation.

It is well established that glutamate and glutamine metabolism is compartmentalized between and within brain cells. This compartmentation is complex and contributes to the remarkable capacity of neurons and astrocytes to discern between exogenous glutamate and endogenous glutamate (reviewed in [53], references therein for details). Strong evidence indicates that exogenous glutamate follows a different metabolic fate than endogenous glutamate. Exogenous glutamate taken up from the extracellular space may be metabolized via transamination catalysed by asparate aminotransferase (AAT) in neurons and isolated nerve endings [54]. AAT can mediate the entrance of the glutamate carbon skeleton into the TCA cycle by converting glutamate to α-ketoglutarate. On the other hand, endogenous glutamate generated from glutamine via glutaminase has been shown to enter the TCA cycle mainly via GDH [53, 54]. Interestingly, in cultured glutamatergic cerebellar neurons, it has been shown that the endogenous neurotransmitter glutamate generated from glutamine via PAG can enter the TCA cycle via AAT [55].Our data show an increased production of endogenous [U-^13^C] glutamate synthesised from [U-^13^C] glutamine consistent with an increased PAG expression in the FTD neurons. Based on the suggested glutamate compartmentation, it can be speculated that in our FTD neuron model system, the observed subsequent increase in ^13^C-labeled α-ketoglutarate after [U-^13^C] glutamine metabolism is to a larger extent derived from AAT activity than from GDH activity. This is consistent with the increased AAT expression found in the FTD neurons (GOT, Fig 1 c2). In contrast, we observed decreased ^13^C-labeled α-ketoglutarate after metabolism of exogenous [U-^13^C] glutamate, which could directly be attributed to decreased GDH expression and, hence, activity. Our results further support the notion of a complex glutamate metabolism compartmentation in neurons as extensively demonstrated previously [53].

Taken together, our findings demonstrate disruption of energy metabolism at the cellular level in hiPSC-derived neurons and astrocytes from FTD3 patients. We show that exquisitely sensitive glutamate-glutamine homeostasis is profoundly affected, implicating the functional involvement of PAG, GDH and GS in FTD3 pathology. These findings point to novel avenues of exploration for future neurodegenerative disease therapies, focusing on the key enzymes catalysing glutamate-glutamine metabolism.

## Methods

### Ethical compliance statement

The Ethics Committee of the Capital Region of Denmark approved the study (H-4-2011-157), and written informed consent was obtained from each participant before enrolment.

### Human iPS cell culture and differentiation into Neurons & Astrocytes

Human dermal fibroblasts cultures were obtained from skin biopsies from two symptomatic (H150, H151) and one pre-symptomatic (H242) FTD3 individuals (all from the same family). Those were expanded as primary fibroblasts and reprogrammed using non-integrative episomal plasmids [56]. All three participants carry a heterozygous mutation with G-to-C transition in the 5′acceptor splice site of exon 6 in *CHMP2B* on chromosome 3^12^. Basic characterization and generation of gene-corrected isogenic controls was achieved via the CRISPR-Cas9 system described in our previous study (H150 ISO, H151 ISO and H242 ISO) [14].

hiPSCs were cultured with ESC medium consisting of Essential 8™ basal medium with supplement (Thermo Fisher Scientific, A1517001) and passaged 1:3–6 with 0.5 mM EDTA on to Vitronectin™-coated (Thermo Fisher Scientific, A14700) dishes.

Neural differentiation of hiPSCs was achieved via a modified dual SMAD protocol and neuronal characterization was performed as described previously [14]. Briefly, iPSC lines were differentiated toward neural-specific progenies employing a modified dual SMAD protocol [57] . Neural induction was initiated by changing the medium to neural basic medium consisted of 50% DMEM/F-12 medium, 50% Neural basal medium (Thermo Fisher Scientific, 10,888–022), N2 (Thermo Fisher Scientific, 17,502–048), B27 without Vitamin A (Thermo Fisher Scientific, 12,587–010), supplemented with dual SMAD inhibitors SB431542 (Selleck, S1067) and LDN193189 (Selleck, S2618). Subsequently, cells were split onto poly-O-Lysine/laminin (Sigma, L2020) for terminal differentiation applying the following media: Neural basic medium supplemented with 20 ng/ml BDNF (Cell Guidance Systems, GFH1–10), 10 ng/ml GDNF (Cell Guidance Systems, GFH2–10), 50 μM db-cAMP (Sigma, D0627) and 200 μM L-Ascorbic acid 2-phosphate (Sigma, A8960). The whole protocol from hiPSC to matured neurons takes 7 weeks.

The initial steps of astrocyte differentiation followed a previously published protocol [58] . Astrocyte progenitors were plated at a seeding density of 50.000 cells/cm^2^ in AMM medium supplemented with 10 ng/ml of Activin A (Thermo Fisher Scientific, PHG9014), 10 ng/ml HRGβ1 (Peprotech, 100–03), 200 ng/ml of IGF1 (Peprotech, 100–11), 1% sodium pyruvate (Thermo Fisher Scientific, 1,136,070), 10% heat-inactivated and toxin-free FBS (Invitrogen) and 200 μM L-Ascorbic acid 2-phosphate (Sigma, A8960). Medium was changed every other day and cells were differentiated for 35+ days. The efficiency of terminal astrocyte differentiation was monitored by assaying for expression of S100β, AQP4 and SOX9 by immunocytochemical staining and RNA seq. In the current study, astrocyte progenitor cells were differentiated for 10 weeks for immunocytochemical analyses and metabolic assays.

### LC-MS/MS-based proteomics

#### Sample collection and protein isolation

Neurons were collected on ice in phosphate-buffered saline (PBS: 137 mM NaCl, 2.7 mM KCl, 7.3 mM Na_2_HPO_4_, 0.9 mM CaCl_2_ and 0.5 mM MgCl_2_, pH 7.4) with protease inhibitor (Complete tablets, Roche). Cell pellets were dissolved in lysis buffer consisting of 6 M urea (Sigma), 2 M thiourea (Sigma), 20 mg/ml sodium dodecyl sulfate (SDS, GE Healthcare), 40 mM N-ethylmaleimide (NEM, Sigma) and protease inhibitor followed by sonication on ice and incubation for 30 min at room temperature (RT).

Following methanol-chloroform precipitation, proteins were denatured and reduced in 6 M urea, 2 M thiourea, and 10 mM tris(2-carboxyethyl) phosphine (TCEP, ThermoFisher). After vortexing, the samples were incubated for 2 h with 1 μl endoproteinase Lys-C (Wako) at RT. The samples were diluted 10 times in 20 mM TCEP in 20 mM triethylammonium bicarbonate (TEAB, Sigma) buffer, pH 7.5 and sonicated on ice, followed by digestion with 1 μg trypsin (Sigma) per 50 μg peptide overnight (O/N) at RT. Protein concentration was measured by Qubit® (Thermo Scientific) according to the manufacturer’s instructions. Seventy microgram of each sample was labeled with Tandem Mass Tag (TMT) 10plex Isobaric label Reagents (Thermo Scientific) according to the manufacturer’s instructions. The labeled peptides were mixed 1:1:1:1:1:1 and dried.

### Hydrophobic interaction liquid chromatography (HILIC) and high pH fractionation

To reduce sample complexity, peptides were fractionated using HILIC as previously described [59]. Samples were diluted in 0.1% TFA and approximately 50 μg peptide was fractioned. Samples were then dissolved in 90% ACN, 0.1% TFA (solvent B) and loaded onto a packed TSKgel Amide-80 (Tosoh Bioscience) micro-capillary column (450 m OD × 320 m ID × 17 cm) using an Agilent 1200 Series HPLC (Agilent). Peptides were separated using a gradient from 100 to 60% solvent B (A = 0.1% TFA) running for 30 min at a flow-rate of 6 μl/min. Fractions were collected every 1 min and combined into 12–15 final fractions based on the UV chromatogram, and subsequently dried by vacuum centrifugation.

To increase the coverage of the peptides, high pH fractionation was also performed using approximately 50 μg peptide. Briefly, the sample was dissolved in 1% ammonium hydroxide (NH3, Sigma), pH 11, and loaded on a R2/R3 column equilibrated with 0.1% NH3. The peptides were eluted in a stepwise fashion using a gradient of 5–60% ACN/0.1% NH3. All fractions were dried by vacuum centrifugation and stored at − 20 °C.

### Reversed-phase NanoLC-ESI-MS/MS

Samples were resuspended in 0.1% formic acid (FA) and loaded onto a two-column EASY-nLC system (Thermo Scientific). The pre-column was a 3 cm-long fused silica capillary (100 μM inner diameter) with a fitted end and packed with ReproSil - Pur C18 AQ 5 μm whereas the analytical column was a 17 cm-long fused silica capillary (75 μm inner diameter) and packed with ReproSil-Pur C18 AQ 3 μm reversed-phase material (both resins from Dr. Maisch Ammerbuch-Entringen).

Peptides were eluted with an organic solvent gradient from 100% phase A (0.1% FA) to 34% phase B (95% ACN, 0.1% FA) at a constant flow-rate of 250 nL/min. Depending on the samples based on the HILIC, the gradient was from 1 to 30% solvent B in 60 min or 90 min, 30 to 50% solvent B in 10 min, 50–100% solvent B in 5 min and 8 min at 100% solvent B.

The nLC was connected online to a QExactive HF Mass Spectrometer (Thermo Scientific) operated at positive ion mode with data-dependent acquisition. The Orbitrap acquired the full MS scan with an automatic gain control (AGC) target value of 3 × 10^6^ ions and a maximum fill time of 100 ms. Each MS scan was acquired at high-resolution (120,000 full-width half-maximum (FWHM)) at m/z 200 in the Orbitrap with a mass range of 400–1400 Da. The 12 most abundant peptide ions were selected from the MS for higher energy collision-induced dissociation (HCD) fragmentation (collision energy: 34 V). Fragmentation was performed at high resolution (60,000 FWHM) for a target of 1 × 10^5^ and a maximum injection time of 60 ms using an isolation window of 1.2 m/z and a dynamic exclusion. All raw data were viewed in Thermo Xcalibur v3.0.

### Proteomic data analysis

Raw data were processed using Proteome Discoverer (v2.1, ThermoFisher) and searched against the Swissprot human database using an in-house Mascot server (v2.3, Matrix Science Ltd.) and the Sequest HT search engine. Database searches were performed with the following parameters: precursor mass tolerance of 10 ppm, fragment mass tolerance of 0.03 Da (HCD fragmentation) and a maximum of 2 missed cleavages for trypsin. All identified peptides were filtered against a Decoy database using Percolator resulting in a false discovery rate (FDR) of 0.01 (FDR < 0.01). Only peptides with Mascot rank 1 and cut-off value of Mascot score > 15 and a SEQUEST HT ∆Cn of 0.1 were considered for further analysis.

Quantitation of identified peptides was performed on Log2-values of measured signal to noise (S/N) values and the data were normalized based on the median. Quantification of proteins was obtained by merging unique non-modified peptides using the R Rollup function (using the DanteR package) including at least two unique peptides per protein and using the mean of the S/N. The intensity factors for the experimental conditions for each peptide or protein were subtracted.

Significantly-regulated proteins were defined via a combination of limma testing and rank products [60] using the crosstalk.bmb.sdu.dk publicly-accessible app, with a *q*-value of 0.05 for significantly-regulated proteins.

### Fluorescent immunocytochemistry and confocal microscopy

For immunofluorescence staining, cells were fixed with 4% formaldehyde in PBS solution for 15 min, permeabilized with 1% Triton X-100 in PBS solution for 15 min and blocked with 2% bovine serum albumin in PBS solution for 1 h. Thereafter, cells were incubated with the following primary antibodies overnight at 4 °C: anti-MAP 2 (1:500; Sigma, M1406), anti-TUJ1 (1:500; Covance, MRB-435P), anti-TAU (1:200; Dako, A0024) and anti-CTIP2 (1:200; Abcam, ab28448). Primary antibodies were detected using secondary antibodies conjugated to Alexa Fluor 488 (1:500; Molecular Probes) and Alexa Fluor 594 (1:500; Molecular Probes). Cells were washed in PBS and nuclei counterstained with DAPI (Sigma-Aldrich, D9542). Finally, coverslips of growing cells were transferred onto glass slides with mounting medium (Dako, S3023) and imaged immediately using sequential line scanning with a Leica TCS SP5 II inverted confocal microscope.

### Incubation experiments using ^13^C-labeled substrates

Stable isotope ^13^C and gas chromatography coupled to mass spectrometry can be used to obtain information regarding metabolic processes as the natural abundance of ^13^C is scarce and, accordingly, the sensitivity for detection of endogenous metabolites is limited. This low natural abundance allows for exogenous employment of ^13^C-labeled substrates to study the significance of diverse metabolic pathways. Therefore, in order to investigate metabolism in cultured neurons and astrocytes, these cells can be incubated in the presence of ^13^C-labelled substrates and the percent ^13^C enrichment determined in various metabolites, thereby mapping the pathways. To achieve this, culture medium was removed and cells were washed twice with 1 ml of PBS. Cell cultures were subsequently incubated for 90 min at 37 °C in 2 ml serum-free culture medium containing 2.5 mM [U-^13^C] glucose (neurons) or in the presence of 250 μM [U-^13^C] glutamate plus 2.5 mM unlabeled glucose (neurons and astrocytes) or 0.5 mM [U-^13^C] glutamine (neurons) plus unlabeled glucose. In order to avoid excitotoxic effects of glutamate in neurons during the incubation period, two glutamate receptor antagonists CNQX (25 μM), a selective antagonist of the α-amino-3-hydroxy-5-methyl-isoxazole-4-propionic acid (AMPA) and kainate receptor subtypes, and D-AP5 (100 μM), an *N*-methyl-D-aspartate (NMDA) antagonist, were also present in the medium [61]. Following incubation, medium was collected and cells were washed with PBS, lysed and extracted with 70% ethanol. Cell extracts were centrifuged at 20,000 g for 20 min (4 °C) to separate the soluble extract (supernatant) from the insoluble components (pellet). Supernatants were lyophilized and reconstituted in water for subsequent biochemical analyses.

### Metabolic mapping using gas chromatography coupled to mass spectrometry (GC-MS)

Extract and media samples were adjusted to pH 1–2 with HCl and evaporated to dryness under nitrogen flow. Analytes were extracted into an organic phase (96% ethanol/benzene) followed by derivatization with 14% DMF/86% MTBSTFA with a modified procedure [62]. Standards containing unlabeled metabolites of interest and cell extracts were separated and analysed in a gas chromatograph (Agilent Technologies 7820A chromatograph, J&W GC column HP-5MS, parts no. 19091S-433) coupled to a mass spectrometer (Agilent Technologies, 5977E). Isotopic enrichment of the metabolites of interest was corrected for natural abundance of ^13^C using the unlabeled standards and calculated according to [63]. Data are presented as labelling (%) of M + X, where M is the mass of the unlabelled molecule and X is the number of labelled C-atoms in a given metabolite.

### Seahorse XFe96 glycolysis assay

Both principal cellular bioenergetic processes - glycolysis and mitochondrial respiration - can be assessed simultaneously with the Seahorse platform (Seahorse Biosciences-Agilent Technologies, USA) in different brain-derived preparations. We have previously demonstrated alterations in mitochondrial function in FTD3 neurons using this approach [14]. Here, glycolytic activity was evaluated by measuring the extracellular acidification rate (ECAR) using a Seahorse XFe96 Extracellular Flux Analyzer (Seahorse Biosciences-Agilent Technologies, USA). Human neuronal progenitor cells cultured with neuronal maturation factors for 1 week were seeded in a Seahorse 96-well cell culture microplate at a density of 6.5 × 10^3^ cells/well. The cells were then allowed to adhere and further mature for 3 to 4 weeks. On the day of the assay, the culture medium was changed for unbuffered DMEM (pH 7.4) supplemented with 2.5 mM glucose and the cells were equilibrated for 10 min at 37 °C in a CO_2_-free incubator. The pH of the reagents used to test mitochondrial function was adjusted to 7.4. The ECAR measurement cycle consisted of 3 min mix and 3 min measurement of the oxygen level. Testing of glycolytic function was evaluated by the sequential injection of the ATP synthesis inhibitor, oligomycin (2 μM final concentration) and a mixture of rotenone (0.5 μM) and antimycin A (0.5 μM) with one ECAR measurement after the first injection and two final measurement cycles. ECAR was simultaneously recorded and calculated by the Seahorse XFe96 software, Wave. Subsequent to the Seahorse analysis, protein content was measured for each well using the Pierce BCA assay with BSA as standard. Based on the ECAR measurements, mitochondrial respiration was calculated by subtracting non-mitochondrial respiration (minimum measurement after rotenone/antimycin injection) from the last measurement obtained before oligomycin injection. Furthermore, we recorded the oxygen consumption rate (OCR) [14]. With these data we were able to calculate the coupling efficiency (in %) as the mitochondrial ATP production rate/ the basal respiration rate × 100 (Seahorse bioscience, Agilent Technologies) [21]. Results are expressed as mean values ± standard error of the mean (S.E.M.), (*n* = 3). The mean values are based on 12 replicates from the three batches of neurons from the three different FTD patient cell lines (H150, H151 and H242).

### Quantitative determination of intracellular amino acids

Amino acids in cell extracts were separated and quantified by reverse-phase high performance liquid chromatography (HPLC) using an Agilent ZORBAX Eclipse plus C18 column (4.6 × 150 mm, particle size 3.5 μm; 959,963–902, Agilent Technologies, Santa Clara, CA, USA), pre-column online o-phthaldialdehyde derivatization and fluorescence detection (338 nm, 10 nm bandwidth, and reference wavelength 390 nm, 20 nm bandwidth). An Agilent 1260 Infinity system coupled to a 1260 Infinity fluorescence detector (Agilent Technologies) was employed. To elute the amino acids, a mobile phase gradient consisting of a mixture of mobile phase A (10 mM Na_2_HPO_4_: 10 mM Na_2_B_4_O_7_, pH 8.2; 5 mM NaN_3_) and mobile phase B (acetonitrile 45%: methanol 45%: water 10%, v:v:v) was used at a flow of 1.5 ml/min with a total run time of 35 min. The amount of amino acids in the samples was quantified based on standards containing a mixture of the amino acids of interest at increasing known concentrations and injected in parallel with the samples [40].

### Western blot analyses

Cell pellets were lysed in lysate buffer (mM: 150 NaCl, 10 Tris base, 1 EDTA and 1% Triton, pH 8) supplied with cOmplete Protease Inhibitor (Roche, Germany) for 1 h on ice. Proteins were denatured by boiling the sample (5 min) in NuPAGE® LDS Sample Buffer (ThermoFisher Scientific, Carlsbad, CA, USA) with 50 mM DTT. Equal amounts of protein per lane were resolved on a 4–12% Bis–Tris pre-cast gel using the XCell SureLockTM mini-electrophoresis system (ThermoFisher). Proteins were transferred onto a PVDF membrane with constant 30 V for 2 h in NuPAGE® Transfer Buffer (ThermoFisher) with 20% methanol. The membranes were blocked for 1.5 h at room temperature (RT) in 2% (w/v) non-fat skim milk protein and 0.1% Tween-20 in wash buffer (mM: 50 Tris base, 100 NaCl, pH 7.2) and were incubated overnight at 4 °C with antibodies against PAG (1:1000; Abcam, ab93434), GDH2 (1:1000; made and kindly gifted by Ioannis Zaganas [64],, GS (1:1000, Abcam, ab73593), GAPDH (1:5000, Santa Cruz biotech, sc-25,778) or β-actin (1:2000; Abcam, ab8227). Following three 5-min washes, the membranes were incubated (1.5 h, RT) with appropriate HRP-conjugated secondary antibodies (Dako, Glostrup, Denmark). All antibodies were diluted in washing buffer with 0.2% (w/v) non-fat skim milk and 0.05% Tween-20. The bands were visualized using ECL™ Prime Western Blotting Detection Reagent (GE Healthcare, Buckinghamshire, UK).

### Data and statistical analysis

Data are presented as mean ± S.E.M. of hiPSC-derived neurons or astrocytes from three different FTD3 patient cell lines (H150, H151 and H242) and their respective isogenic controls. In mass spectrometry and western blot analyses, three to four technical replicates per cell line were used for each condition tested. For the ^13^C-labeling studies, data are depicted as labeling (%) of M + X, where M is the mass of the unlabeled metabolite and X is the number of ^13^C-labeled carbon atoms. Statistical evaluations comprised unpaired multiple *t* test followed by Bonferroni-Dunn post hoc test to correct for multiple comparisons or two-way ANOVA correcting for multiple comparisons (Sidak post hoc test) as indicated in figure legends or throughout the text. Data are assumed to be sampled from populations with the same scatter. Analyses were performed using *GraphPad Prism 7.0*. *P* values below 0.05 were considered statistically significant.

## Supplementary information

**Additional file 1: Figure S1.** **a, b** Differentially expressed canonical pathways identified by Ingenuity Pathway Analysis of the RNA-seq data [14] and quantitative proteomics data. “Canonical pathways” are defined by a cluster of related signature genes, which constitutes the ratio’s denominator. The numerator is the number of signature genes that were significantly changed in the transcriptomics or proteomics data set. *P*-value is the probability that the ratio occurred by chance.

**Additional file 2: Supplementary methods.** Quantitative determination of TCA cycle metabolite amounts and [^13^C] labeled metabolite isotopologues by UHPLC-ESI-MS/MS. The quantification of the total amounts of succinate and malate was performed employing a Bruker Advance UHPLC system coupled to ESI-MS/MS (EVOQTQMS) for chromatographic separation, fragmentation and quantification as described in Larsen (*Larsen, T.M. and Stürup, S. 2019. Development of an UHPLC-ESI-MS/MS method for simultaneous quantification of isotope labeled metabolites and investigation of their fragmentation patterns, University of Copenhagen, Denmark*) The column used to separate the analytes was a Phenomenex Luna® Omega, 1.6 μm Polar C18, 100 × 2.1 mm thermostated at 30 °C with a Phenomenex SecurityGuardTM ULTRA Cartridge precolumn. For separation, a mobile phase consisting of 15 mM glacial acetic acid in water and a mobile phase B of pure methanol were employed. The following gradient elution was applied: 0–5 min; 2% B flow rate 300 μL/min, 6–8 min; 100% B flow rate 400 μL/min, 9–11 min; 2% B flow rate 300 μL/min. Quantification of isotopologues in samples was performed with Single Ion Monitoring (SIM) scan whereas the fragmentation by tandem mass spectrometry (MS/MS) was performed with product ion scan. Matrix mathematics was used to calculate the abundance/quantity of each isotopologue from the data obtained from the MS analysis. Only two intermediates, succinate and malate, were detected due to the detection limit of the UHPLC-ESI-MS/MS system.

## Data Availability

Please contact author for data requests.
