# Supplementary figures and images for: Glutamate-glutamine homeostasis is perturbed in neurons and astrocytes derived from patient iPSC models of frontotemporal dementia
Source: Mol Brain. 2020 Sep 14;13:125. doi: 10.1186/s13041-020-00658-6 (PMC7491073; doi:10.1186/s13041-020-00658-6)

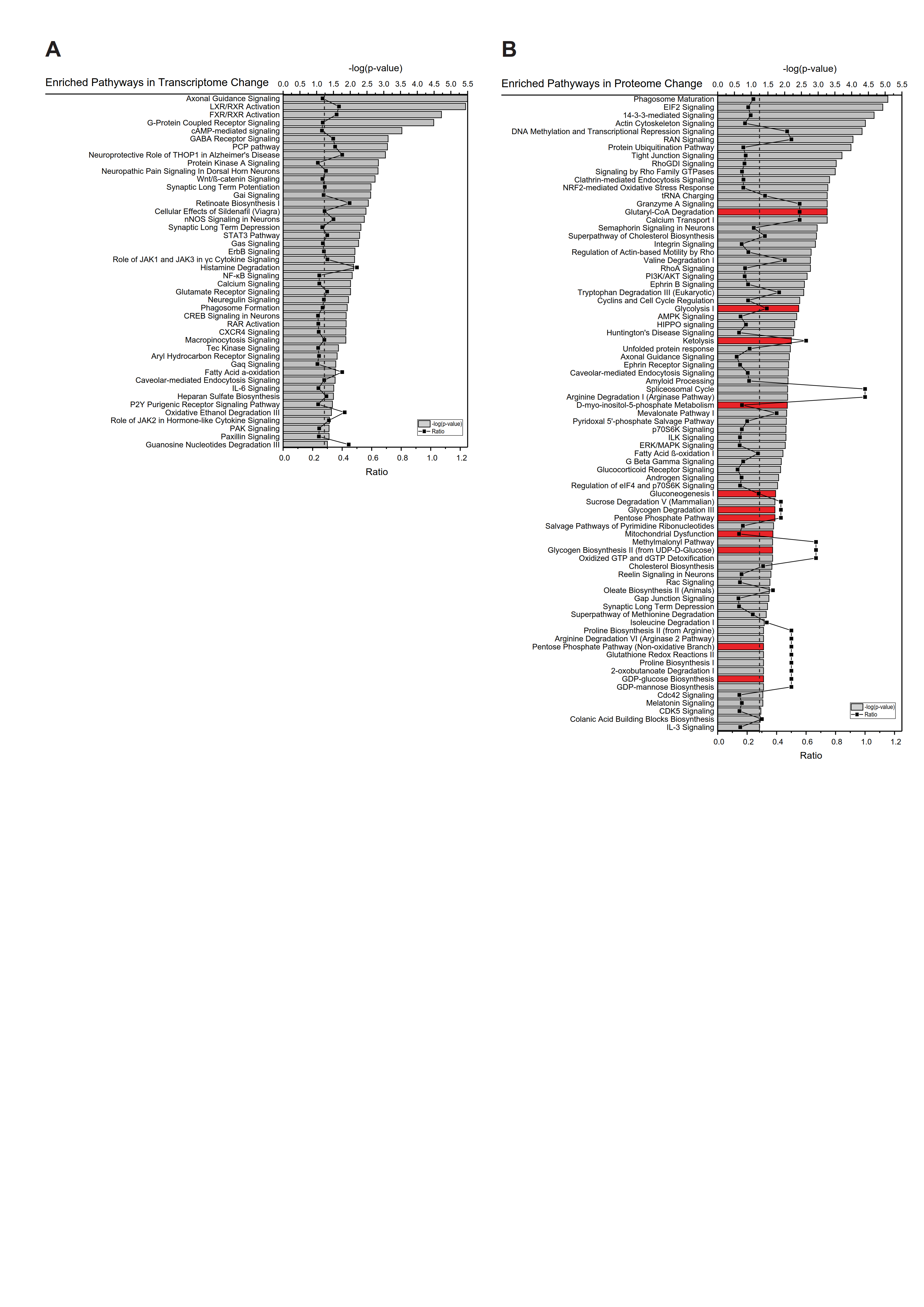

Supplement: Supplementary file 1 — Additional file 1: Figure S1. a, b Differentially expressed canonical pathways identified by Ingenuity Pathway Analysis of the RNA-seq data [14] and quantitative proteomics data. “Canonical pathways” are defined by a cluster of related signature genes, which constitutes the ratio’s denominator. The numerator is the number of signature genes that were significantly changed in the transcriptomics or proteomics data set. P-value is the probability that the ratio occurred by chance. [file 13041_2020_658_MOESM1_ESM.tif]
