# Supplementary material for: Glutamate-glutamine homeostasis is perturbed in neurons and astrocytes derived from patient iPSC models of frontotemporal dementia
Source: Mol Brain. 2020 Sep 14;13:125. doi: 10.1186/s13041-020-00658-6 (PMC7491073; doi:10.1186/s13041-020-00658-6)
Supplement: Supplementary file 2 — Additional file 2: Supplementary methods. Quantitative determination of TCA cycle metabolite amounts and [13C] labeled metabolite isotopologues by UHPLC-ESI-MS/MS. The quantification of the total amounts of succinate and malate was performed employing a Bruker Advance UHPLC system coupled to ESI-MS/MS (EVOQTQMS) for chromatographic separation, fragmentation and quantification as described in Larsen (Larsen, T.M. and Stürup, S. 2019. Development of an UHPLC-ESI-MS/MS method for simultaneous quantification of isotope labeled metabolites and investigation of their fragmentation patterns, University of Copenhagen, Denmark) The column used to separate the analytes was a Phenomenex Luna® Omega, 1.6 μm Polar C18, 100 × 2.1 mm thermostated at 30 °C with a Phenomenex SecurityGuardTM ULTRA Cartridge precolumn. For separation, a mobile phase consisting of 15 mM glacial acetic acid in water and a mobile phase B of pure methanol were employed. The following gradient elution was applied: 0–5 min; 2% B flow rate 300 μL/min, 6–8 min; 100% B flow rate 400 μL/min, 9–11 min; 2% B flow rate 300 μL/min. Quantification of isotopologues in samples was performed with Single Ion Monitoring (SIM) scan whereas the fragmentation by tandem mass spectrometry (MS/MS) was performed with product ion scan. Matrix mathematics was used to calculate the abundance/quantity of each isotopologue from the data obtained from the MS analysis. Only two intermediates, succinate and malate, were detected due to the detection limit of the UHPLC-ESI-MS/MS system. [file 13041_2020_658_MOESM2_ESM.docx]

**Supplementary methods.**

Quantitative determination of TCA cycle metabolite amounts and [^13^C] labeled metabolite isotopologues by UHPLC-ESI-MS/MS. The quantification of the total amounts of succinate and malate was performed employing a Bruker Advance UHPLC system coupled to ESI-MS/MS (EVOQTQMS) for chromatographic separation, fragmentation and quantification as described in Larsen (*Larsen, T.M. and Stürup, S. 2019. Development of an UHPLC-ESI-MS/MS method for simultaneous quantification of isotope labeled metabolites and investigation of their fragmentation patterns, University of Copenhagen, Denmark*) The column used to separate the analytes was a Phenomenex Luna® Omega, 1.6 µm Polar C18, 100 x 2.1 mm thermostated at 30 ºC with a Phenomenex SecurityGuardTM ULTRA Cartridge precolumn. For separation, a mobile phase consisting of 15 mM glacial acetic acid in water and a mobile phase B of pure methanol were employed. The following gradient elution was applied: 0-5 min; 2 % B flow rate 300 µL/min, 6-8 min; 100 % B flow rate 400 µL/min, 9-11 min; 2 % B flow rate 300µL/min. Quantification of isotopologues in samples was performed with Single Ion Monitoring (SIM) scan whereas the fragmentation by tandem mass spectrometry (MS/MS) was performed with product ion scan. Matrix mathematics was used to calculate the abundance/quantity of each isotopologue from the data obtained from the MS analysis. Only two intermediates, succinate and malate, were detected due to the detection limit of the UHPLC-ESI-MS/MS system.
